# Identification and Functional Characterization of a Tonoplast Dicarboxylate Transporter in Tomato (*Solanum lycopersicum*)

**DOI:** 10.3389/fpls.2017.00186

**Published:** 2017-02-16

**Authors:** Ruiling Liu, Boqiang Li, Guozheng Qin, Zhanquan Zhang, Shiping Tian

**Affiliations:** ^1^Key Laboratory of Plant Resources, Institute of Botany, Chinese Academy of SciencesBeijing, China; ^2^College of Life Sciences, University of Chinese Academy of SciencesBeijing, China

**Keywords:** tomato, tonoplast transporters, overexpression, RNAi, organic acid composition

## Abstract

Acidity plays an important role in flavor and overall organoleptic quality of fruit and is mainly due to the presence of organic acids. Understanding the molecular basis of organic acid metabolism is thus of primary importance for fruit quality improvement. Here, we cloned a putative tonoplast dicarboxylate transporter gene (*SlTDT*) from tomato, and submitted it to the NCBI database (GenBank accession number: KC733165). SlTDT protein contained 13 putative transmembrane domains *in silico* analysis. Confocal microscopic study using green fluorescent fusion proteins revealed that SlTDT was localized on tonoplast. The expression patterns of *SlTDT* in tomato were analyzed by RT-qPCR. The results indicated that *SlTDT* expressed in leaves, roots, flowers and fruits at different ripening stages, suggesting *SlTDT* may be associated with the development of different tissues. To further explore the function of *SlTDT*, we constructed both overexpression and RNAi vectors and obtained transgenic tomato plants by agrobacterium-mediated method. Gas chromatography-mass spectrometer (GC-MS) analysis showed that overexpression of *SlTDT* significantly increased malate content, and reduced citrate content in tomato fruit. By contrast, repression of *SlTDT* in tomato reduced malate content of and increased citrate content. These results indicated that SlTDT played an important role in remobilization of malate and citrate in fruit vacuoles.

## Introduction

Organic acids together with sugars are the main metabolites in ripe fruits and contribute to fruit flavor (Etienne et al., 2013; Zhu et al., 2013). Malate is the predominant organic acid in many fruits including tomato (Kortstee et al., 2007), apple (Liu et al., 2016), pear (Chen et al., 2007), peach (Moing et al., 2000), and loquat (Chen et al., 2009), while citrate is dominant in citrus (Sadka et al., 2000) and mango (Gil et al., 2000). Malate and citrate fulfill a wide range of important functions, the most universal one being their role in tricarboxylic acid (TCA) cycle as respiratory substrate, for production of ATP and NADH (Emmerlich et al., 2003; Fernie and Martinoia, 2009). They are also involved in many processes, such as osmotic pressure maintenance, pH and stomatal regulation, amino acid biosynthesis, and resistance to heavy metal toxicity (Schulze et al., 2002; Centeno et al., 2011).

Previous studies have reported that malate is produced in the cytoplasm and then transport into the central vacuole, which occupies as much as 90% of the total mature cell volume (Yamaki, 1984; Sweetman et al., 2009). The concentration of malate in the cytoplasm is maintained constant by transporting excess malate into the vacuole, so that it doesn’t affect the cellular activities in cytosol (Emmerlich et al., 2003). But this traffic is not all one-way. As fruit ripens, malate would leave the vacuole to be re-metabolized in the cytoplasm, with needs other solutes moving back into the vacuole to compensate (Sweetman et al., 2009). Toward this end, a better understanding of malate transport across tonoplast is of great importance.

To facilitate the exchange of organic acid across the tonoplast, plant vacuoles are equipped with a multitude of channels or transporters either by facilitated diffusion or by active transport mechanism (Terrier et al., 2001; Hafke et al., 2003; Shiratake and Martinoia, 2007; Etienne et al., 2013). In *Arabidopsis thaliana*, there is a well characterized tonoplast dicarboxylate transporter (AttDT), which is a homolog to the human sodium/dicarboxylic co-transporter (Emmerlich et al., 2003; Hurth et al., 2005). Subsequently, members of the aluminum-activated malate transporter (ALMT) gene family (*AtALMT9* and *AtALMT6*) were identified to encode a vacuolar malate channel in mesophyll and guard cells (Kovermann et al., 2007; Meyer et al., 2011). Despite the importance of malate in tomato (Centeno et al., 2011), the mechanisms involved in its intracellular compartmentation was not clear. In an effort to decipher the function of tomato ripening-associated membrane protein (TRAMP), Chen et al. (2001) observed that antisense inhibition of this channel protein increased acids and decreased sugars in tomato fruit. They also proposed that TRAMP were involved in the movement of organic acids and/or sugars between the vacuole and cytosol, but the specific molecular mechanism remains unclear. Recently, two putative malate transporter genes (*SlALMT4* and *SlALMT5*) were found to be highly expressed in tomato fruit (Sasaki et al., 2016). One of them, SlALMT5, localized to the endoplasmic reticulum (ER) and transported malate and inorganic anions such as nitrate and chloride, but not citrate. What’s more, overexpression of *SlALMT5* in tomato substantially improved the contents of malate and citrate in mature seeds, but not in fruits (Sasaki et al., 2016).

Although some transporters have been identified in tomato, much research is still needed to identify and fully characterize the mechanisms that transport citrate and malate across the vacuolar membrane. Here, we described the identification and characterization of a novel tonoplast dicarboxylate transporter gene in tomato fruit. Its subcelluar localization together with expression profiles were analyzed during fruit development stage for detailed functional characterization. To further explore the function of this gene, we obtained both overexpression and RNAi transgenic tomato plants to determine if they produce the predicted changes in the chemical composition of tomato fruit. The information obtained in the present study would not only further our understanding of the physiological mechanisms controls organic acid metabolism in fruit, but also provides a basis for developing new strategies for fruit flavor improvement.

## Materials and Methods

### Plant Material and Growth Conditions

In our experiment, plants of *Solanum lycopersicon* Mill. cv. Ailsa Craig (AC), a near-isogenic tomato line, as the wild type plants. Seeds of tomato were kindly provided by Dr. James J. Giovannoni (Boyce Thompson Institute for Plant Research, Cornell University, Ithaca, NY, USA). The plants were grown in a greenhouse using standard cultural practices, with regular additions of fertilizer and supplementary lighting when required. For plants grown *in vitro*, seeds were germinated on MS medium (Murashige and Skoog, 1962). Conditions in the culture chamber room were set as follows: 16-h-day/8-h-night cycle, 25/18°C day/night temperature, 80% relative humidity. The transgenic plants were grown in peat-based compost supplemented with fertilizer in greenhouses equipped with heating and cooling systems and supplemental lighting. Plants of the first generation (T0) came from tissue culture, and plants of the second generation (T1) were from seedlings. Flowers were tagged at anthesis to determine maturity and ripening-stage. The ripening stages of tomato fruits were divided according to days after anthesis (dpa) and fruit color. Immediately upon harvesting, seeds and locular gel were removed and the pericarp frozen with liquid nitrogen, and stored at 80°C until further use.

### Isolation of *SlTDT*

RNA isolation from pericarp of the fruits was conducted using the method described by Moore et al. (2005). Then, 1 mg of total RNA was treated with DNase I and used to synthesize first-strand cDNA through reverse transcription-PCR using TransScript First-Strand cDNA Synthesis SuperMix (TransGen Biotech) with tailed oligo(dT)18 primer. A full-length cDNA sequence of *SlTDT* was amplified from fruit cDNA using primers of SlTDT-F (5′-ATGAATGGAGATCATCATGA-3′) and SlTDT-R (5′-CTAGCCACACATATGCAAA-3′) through TransStart FastPfu PCR SuperMix (TransGen Biotech). The amplified products were ligated into a pEASY-Blunt vector and subsequently transformed into *Escherichia coli* Trans1-T1 (TransGen Biotech). Positive colonies were selected and confirmed by sequencing (*BGI*, Beijing, China).

### Phylogenetic Analysis

Sequence alignment of *SlTDT* and tonoplast dicarboxylate transporters from other organisms was performed using ClustalX (version 2.1) software (Livak and Schmittgen, 2001) with default multiple parameters and PAM series protein weight matrix. The alignment was then imported into MEGA (version 5.2) software and the phylogenetic tree was constructed by the neighbor-joining statistical method with 1000 bootstrap replicates.

### Transient Expression of *SlTDT* in Tobacco

To construct the 35S::GFP-*SlTDT*, the full-length *SlTDT* cDNA was cloned into the plant expression vector pCAMBIA2300 downstream of GFP. Transient expression of the GFP-*SlTDT* fusion construct in tobacco was performed as described with slight modifications (Batoko et al., 2000). Individual *Agrobacterium* colonies were cultured at 28°C to the stationary phase, washed, and resuspended in infiltration medium containing 10 mM MES (pH 5.6), 10 mM MgCl_2_, and 200 mM acetosyringone to an OD 600 of 0.5. Then the suspension was injected into the abaxial epidermis of leaves of 5–6-week-old *Nicotiana tabacum* plants using a needleless syringe. Plants were incubated for 2–3 days at 25°C under constant light and transformed leaves were used to extract protoplasts and vacuoles for confocal microscopy.

### Vector Constructs and Tomato Transformation

To generate the *SlTDT* overexpression (OE) construct, the full-length cDNA (nucleotides 1-1659) was inserted into the plant binary vector pBI121 containing the cauliflower mosaic virus 35S promoter. To construct the RNA interference (RNAi) plasmid, a 363 bp *SlTDT* fragment was amplified from tomato cDNA using the primer pair: 5′-GTGCTGGTGATGGAACTG-3′ and 5′-GTAGGAGGGGATGGATGT-3′. The resulting product was cloned into the PCR8/GW/TOPO Gateway entry vector (Invitrogen). The cloned fragment was subsequently transferred into the destination vector pK7GWIWG2 (Karimi et al., 2002) using the LR Clonase II enzyme (Invitrogen). All constructs generated were confirmed by sequencing using universal primers (BGI, Guangzhou, China).

The above constructs were transformed into *Agrobacterium tumefaciens* GV3101 by electroporation, respectively, and *Agrobacterium*-mediated transformation was performed following the method described previously (Meissner et al., 1997, 2000). The hardened putative transgenics were shifted to the greenhouse and confirmed by PCR analysis using primers NPTII-F (5′-TCGGCTATGACTGGGCACAACAG-3′) and NPTII-R (5′-GGCGATACCGTAAAGCACGAGGAA-3′). Three independent transformants (T0) were identified by their ability to grow on kanamycin and the transcript levels of *SlTDT* were analyzed by RT-qPCR. Transformed seeds of T0 plants were selected on half MS medium containing 100 mg L^-1^ kanamycin. Kanamycin-resistant plants (T1) were transferred to soil and grown to maturity in a phytotron (22°C, 16 h light/8 h dark photoperiod) and used for phenotype analysis.

### RT-qPCR Analysis

For expression analysis in different tissues, total RNA was also extracted from roots, stems, young leaves, mature leaves and flowers, respectively, using the RNAiso reagent (TaKaRa, Japan), then used as the template for reverse-transcribed via PrimeScript RT reagent Kit with gDNA Eraser (TaKaRa Corp., Dalian, P.R. China). Real-time quantitative PCR (RT-qPCR) assays was performed using SYBR Premix Ex Taq (TaKaRa Corp., Dalian, P.R. China) and the StepOne Plus Real-time PCR system (Applied Biosystems, Foster City, CA, USA). All PCR primers (Supplementary Table S1) were designed using Primer Express 3.0 (Applied Biosystems, Foster City, CA, USA), and primer specificity was determined by melting curve analysis. The relative expression levels were estimated using the 2^-ΔΔCt^ method (Livak and Schmittgen, 2001). β-Actin (Accession number: AB35991) was used as the endogenous control for quantifying mRNA levels. Three independent biological replicates were analyzed for each sample.

### Metabolite Analysis

Metabolite analysis by Gas chromatography-mass spectrometer (GC-MS) was carried out according to a reported method (Batoko et al., 2000). with minor modifications to assay the changes in metabolites among the samples. Peel of 15 T1 tomato fruit and flesh (300 mg) of wild type was homogenized individually using a ball mill pre-cooled with liquid nitrogen and extracted in 2700 μL of methanol; 300 μL of internal standard (0.2 mg/mL ribitol) was subsequently added as a quantification standard. The mixture was extracted for 15 min at 70°C and centrifuged at 2200 rpm. The upper (methanol) phase was removed, concentrated and dried in a vacuum. The residue was dissolved in 50 μL of 20 mg/mL pyridine hydrochloride, and incubated for 30 min at 50°C. The sample was treated with 50 μL of BSTFA (1% TMCS) for 40 min at 60°C.

Exactly 1 μL of each sample was accurately absorbed and injected into the gas chromatograph through a fused-silica capillary column (30 m × 0.25 mm × 0.25 μm; DB-5 MS stationary phase). The injector temperature was 250°C and the carrier gas had a flow rate of 1.0 mL/min. The column temperature was held at 100°C for 1 min, increased to 184°C at a temperature gradient of 3°C /min, increased to 190°C at 0.5°C /min, held for 1 min, increased to 280°C at 15°C/min and then held for 5 min. Helium (purity 99.999%) was used as the carrier gas with a flow rate of 1 mL/min. The significant MS operating parameters were as follows: ionization voltage was 70 Ev (electron impact ionization), ion source temperature was 200°C and the interface temperature was 250°C. TIC (total ion current) spectra were recorded in the mass range of 45–600 atomic mass units (amu) in scanning mode.

## Results

### Cloning of a Full-Length cDNA of the *SlTDT* Gene and Sequence Analysis

**FIGURE 1:**
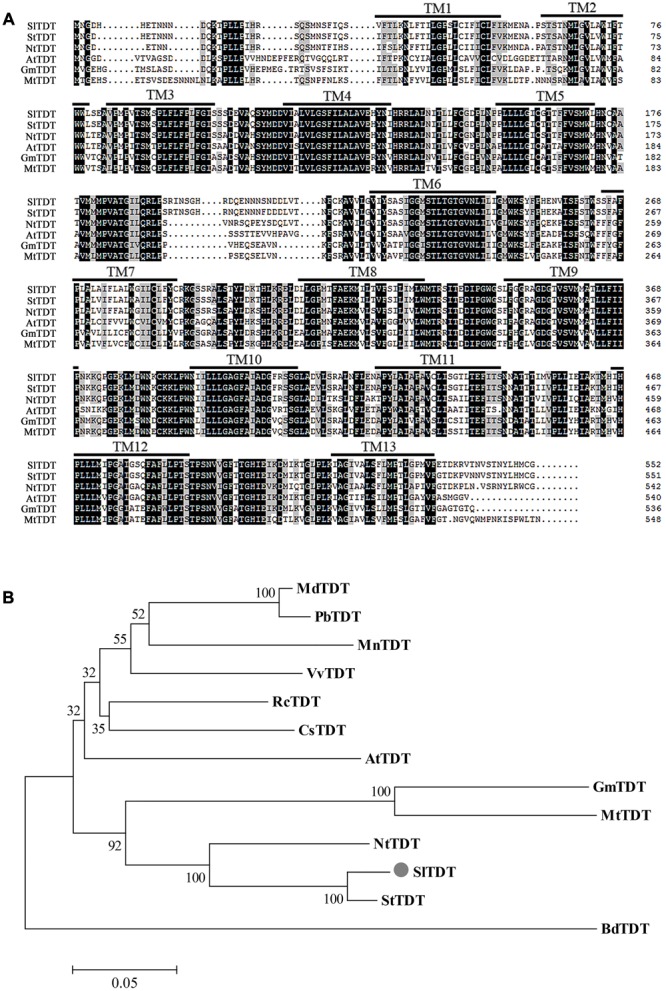
**Sequence analysis of *SlTDT* (A)** Alignment of the deduced amino acid sequences of *SlTDT* with TDT proteins from other species by ClustalX. Identical and conserved residues are shaded in black and gray, respectively. The black lines depict the 13 transmembrane domains (TM) predicted by the TMHMM program (www.cbs.dtu.dk/services/TMHMM-2.0/). **(B)** Phylogenetic tree of amino acid sequences of TDT from tomato and other plant species. Sequences were aligned using the ClustalW multiple sequence alignment program. The phylogenetic tree was generated using the neighbor-joining method. Scale above the tree represents branch length measured by the number of amino acid replacements per position. Bootstrap values are shown for each branch as a percentage of 1000 replicates. Bar, 0.05. GenBank accession numbers of proteins used for sequences and phylogenetic analysis is as follows: StTDT (XM_006352290.20, NtTDT (XM_009630263.1), AtTDT (NM_124129.3), GmTDT (XM_003534797.3), MtTDT (XM_003610484.2), MdTDT (XM_008372780.1), RcTDT (XM_002531562.2), VvTDT (XM_010647887.1), BdTDT (XM_003578273.3), CsTDT (NM_001288949.1), PbTDT (XM_009354144.1).

**FIGURE 2:**
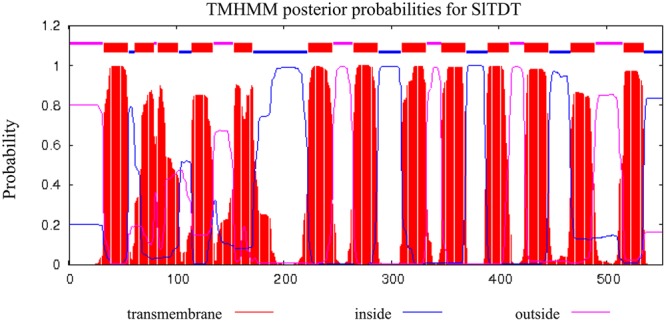
**Hydropathy profiles and membrane-spanning models of *SlTDT.*** The calculation was performed using an algorithm published by Krogh et al. (2001). Blocks, lower lines, and upper lines at the top of each profile indicate putative transmembrane domains, peptides facing the inside of the membrane, and peptides facing the outside of the membrane, respectively.

To identify these unknown vacuolar-transporter-like sequences, we performed a Blast search against the database of tomato with the sequences of *AtTDT* (AT5G4756). We found that the tomato genome contains a single copy of an ORF (Solyc11g012360) exhibiting highest homologies with its *Arabidopsis* counterpart. The corresponding cDNAs comprising the entire ORF were amplified from the cDNA, which has been deposited in GenBank (accession No. KC733165). The putative protein comprising 532 amino acid in length, is predicted to contain 13 transmembrane domains, and possesses a calculated molecular mass of 60.6 kDa and an isoelectric point (pI) of 7.18 (**Figure 1A**). Sequence analysis revealed that the deduced amino acid sequence of *SlTDT* shared roughly 96, 83, and 63% identity with homologous sequences from potato (*StTDT*, XM_006352290.2), tobacco (*NtTDT*, XM_009630263.1), and *A. thaliana* (*AtTDT*, NM_124129.3), respectively (**Figure 1A**). To compare the amino acid similarity between the predicted SlTDT peptides and the TDTs isolated from other plant species, an unrooted phylogenetic tree was constructed using the program MEGA 5.0 (**Figure 1B**). This result showed that *SlTDT* was most closely related to the functional transporter *StTDT* from potato fruit. Analysis of the deduced amino acid sequences by TMHMM revealed the presence of 13 transmembrane domains (**Figure 2**).

### Subcellular Localization

**FIGURE 3:**
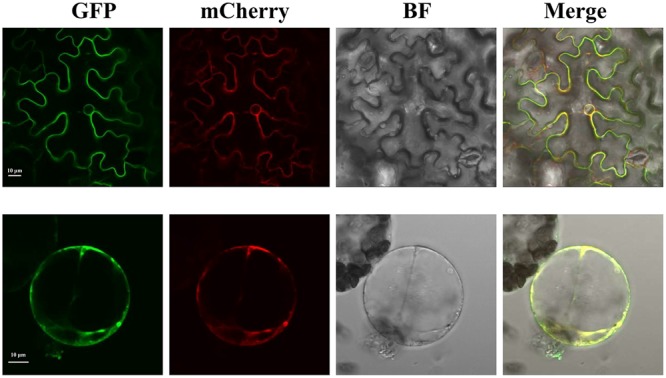
**Confocal images of the subcellular localization of SlTDT-GFP fusions in tobacco leaf mesophyll cell.** The tonoplast marker TIP-RFP was used as the control. The GFP fusion protein is shown in green; the RFP fusion protein is shown in red. All images shown were acquired using the same photomultiplier gain and offset settings. Scale bar: 10 μm.

To investigate the subcellular localization of SlTDT, we transiently expressed a GFP-SlTDT fusion construct under the control of the CaMV35S promoter in the epidermal cells of tobacco leaves. Confocal microscopy of transformed tobacco leaves revealed the vacuolar localization of SlTDT in plant. **Figure 3** shows GFP-SlTDT fluorescence in the stomatal apparatus of tobacco leaves. Clear fluorescence of the fusion protein was found in the tonoplast, labeling the vacuole membrane and not the plasma membrane. In epidermal cells transformed with the control vector pCAMBIA2300-eGFP, green fluorescence was distributed in both the cytoplasm and nucleus of tobacco epidermal cells (**Figure 3**). In contrast, green fluorescence was exclusively detected in the vacuole membrane of cells transformed with the fusion plasmid of pCAMBIA2300eGFP-*SlTDT* (**Figure 3**). These results showed clearly that SlTDT localized to the vacuole membrane.

### Expression Pattern of *SlTDT* in Tomato

**FIGURE 4:**
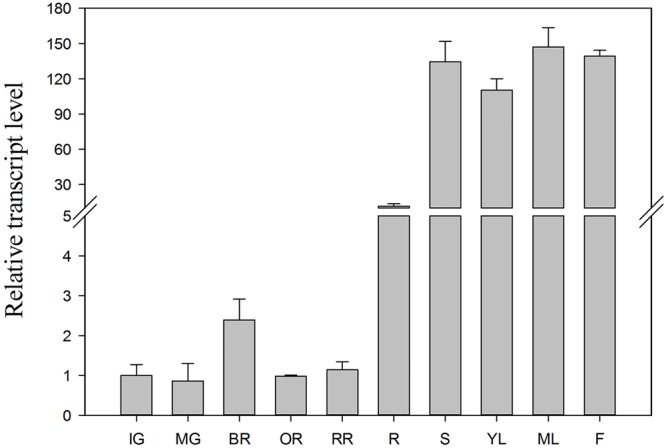
**Transcript levels of *SlTDT* in vegetative and reproductive tomato organs.** Relative expression was normalized using actin as an internal control. Data represent the means ± SD of three replicates. IG, immature green fruits; MG, mature green fruits; BR, breaker fruits; OR, orange fruits; RR, red ripe fruits; R, roots; S, stems; YL, young leaves; ML, mature leaves; F, flowers.

To investigate changes in SlTDT expression level, qRT-PCR was performed using RNA isolated from roots, stems, young leaves, mature leaves, flowers and fruits at different ripening stages (**Figure 4**). Transcript level of *SlTDT* differed among the various organs, with its transcript being most abundant in young leaves, and increased during fruit ripening, and then dropped (**Figure 4**).

### Metabolic Analysis of Transgenic Tomato

To further explore the function of *SlTDT*, a 35S:*SlTDT* overexpression construct and a *SlTDT* RNAi construct were separately introduced into tomato by agrobacterium-mediated method. Three independent transgenic lines corresponding to each construct were identified by their ability to grow on kanamycin. Transcript expression of *SlTDT* in kanamycin-resistant plants (T0) was analyzed by RT-qPCR and two overexpression lines (OE2, OE3) and two RNAi lines (R1, R2) were selected for further study according to their transcript levels of *SlTDT* (Supplementary Figure S1). We observed that the transgenic *SlTDT* T1 plants grown in phytotron showed normal growth phenotype and the size and ripening process of their fruits were similar to those of WT plants (Supplementary Figures S2 and S3).

**FIGURE 5:**
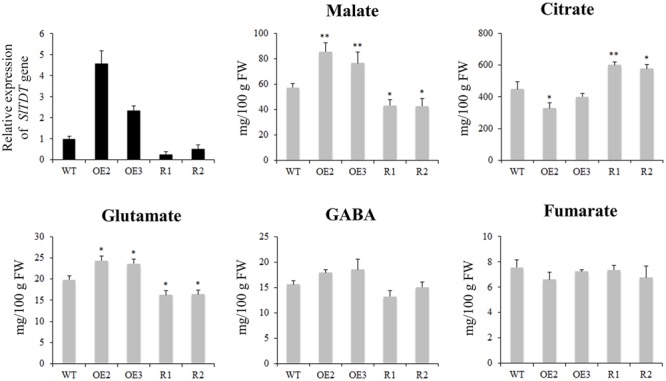
**Expression levels of *SlTDT* gene and contents of organic acid and amino acids in tomato fruit of WT, OE (OE2, OE3) and RNAi (R1, R2) transgenic lines.** Data are means ± SD of three replicates. The level of significance compared with the WT was determined using Student’s *t*-test (^∗^*P* < 0.05, ^∗∗^*P* < 0.01).

GC-MS analysis revealed that fruit of *SlTDT* overexpression lines exhibited higher malate level and lower citrate level at orange ripe stage compared with that of the control. By contrast, silencing of *SlTDT* led to lower malate level and higher citrate level than that in control (**Figure 5**). Moreover, overexpression of *SlTDT* significantly increased the content of glutamate, while repression of *SlTDT* reduced the content of glutamate.

## Discussion

Vacuoles play a critical role in cell turgor regulation, pH and ion homeostasis, cellular detoxification, and defense responses in higher plants (Taiz, 1992; Marty, 1999). Moreover, they contribute to fruit quality by serving as the main reservoir for nutrients and metabolites, such as sugars, organic acids, amino acids, pigments, ions, and water (Shiratake et al., 2000). During fruit development and ripening, all of these functional roles depend on the participation of specific and efficient transport proteins, such as sugar transporters, organic acid transporters, aquaporins, and so on (Martinoia et al., 2000, 2007; Shiratake and Martinoia, 2007). To date, however, only a few tonoplast-localized transporters have been functionally characterized.

Malate is the major organic acid in many fruits, such as peach (Moing et al., 2000), apple (Yamaki, 1984), and tomato (Centeno et al., 2011). Therefore, vacuolar malate accumulation, play an important role in fruit development and might contribute to the final quality of fruit at harvest (Etienne et al., 2013). However, the data on the vacuolar transporters involved in malate accumulation in tomato are not available. Based on previous studies in *A. thaliana* (Emmerlich et al., 2003; Hurth et al., 2005), we hypothesized that tonoplast dicarboxylate transporter could be involved in the vacuolar accumulation of malate in tomato. In this research, we found the tomato genome contains a single copy of an ORF encoding a hydrophobic protein that exhibits high sequence similarity to *AtTDT*. The deduced protein of SlTDT had a predicted topology of 13 transmembrane domains and resides in the subcellular vacuolar membrane. Similarly, it has been proposed that citrate accumulation in citrus was controlled by vacuolar citrate/H^+^ antiporter CsCit1, another homolog of *AtTDT* (Shimada et al., 2006). RT-qPCR analysis indicated that the *SlTDT* gene was expressed in both vegetative and reproductive organs, with its transcript being most abundant in young leaves, and increased during fruit ripening, and then dropped. A possible explanation for this expression pattern could be an involvement of malate in photosynthetic metabolism of leaves (Scheibe, 2004).

Malate transporters might contribute to the acidity of fruit. For instance, VvALMT9 has been proved to be involved in the transport of malate and tartrate to the vacuoles in grape (De Angeli et al., 2013), and malate transporter “Ma” was largely responsible for fruit acidity variations in apple (Bai et al., 2012). In addition, Sasaki et al. (2016) revealed the role of two malate channels in transport of malate across tonoplast, and suggested that one of them SlALMT5 had a great impact on the content of malate and citrate in mature seeds, but not in fruits. To elucidate the effects of transgenic approach on tomato fruit, metabolic changes in fruits were measured. Our results demonstrate that the overexpression of *SlTDT* led to higher malate content and lower citrate content as compared with that of the control. By contrast, repression of *SlTDT* reduced malate content and increased citrate content in tomato fruit. These results were consistent with recent report by Frei (2015), who found that overexpression of *AtTDT* could lead to an increased concentration of malate, meanwhile a reduced concentration of citrate. They also demonstrated that the TDT functioned as an antiporter of malate and citrate, which involved in malate import and citrate export, simultaneously (Frei, 2015). Therefore, we hypothesize that SlTDT might be an antiporter of malate and citrate, which was supported by the changes of citrate content in OE/RNAi transgenic tomato fruit. However, the metabolite level does not change sharply as compared to the wild type, suggesting there may be other transporters or channels responsible for malate/citrate movement across the tonoplast.

Taken together, this study presents the first isolation and functional characterization of SlTDT, a tonoplast dicarboxylate transporter gene, in tomato, and provides further evidence that SlTDT plays an important role in malate and citrate transport. Our findings have important implications for understanding of tonoplast dicarboxylate transporter in organic acid metabolism, which is crucial for fruit flavor.

## Author Contributions

ST conceived and designed the study. RL performed the study. BL and ZZ analyzed the data. RL wrote the first draft of the manuscript. ST and GQ revised the final version of the text. All five authors read and approved the final manuscript.

## Conflict of Interest Statement

The authors declare that the research was conducted in the absence of any commercial or financial relationships that could be construed as a potential conflict of interest.
