# Supplementary material for: Identification and Functional Characterization of a Tonoplast Dicarboxylate Transporter in Tomato (Solanum lycopersicum)
Source: Front Plant Sci. 2017 Feb 16;8:186. doi: 10.3389/fpls.2017.00186 (PMC5311036; doi:10.3389/fpls.2017.00186)
Supplement: Supplementary file 1 [file Table_1.DOCX]

Table S1 Primer sequences used for real-time PCR

| **Gene** | **Forward primer (5’-3’)** | **Reverse primer (5’-3’)** |
| --- | --- | --- |
| *SlTDT* | CGTGCTGGTGATGGAACTGT | TTTCTCCCCTGGTTGCTTTTT |
| *SlActin* | TGTCCCTATTTACGAGGGTTATGC | AGTTAAATCACGACCAGCAAGAT |
