# Supplementary material for: Identification and Functional Characterization of a Tonoplast Dicarboxylate Transporter in Tomato (Solanum lycopersicum)
Source: Front Plant Sci. 2017 Feb 16;8:186. doi: 10.3389/fpls.2017.00186 (PMC5311036; doi:10.3389/fpls.2017.00186)
Supplement: Supplementary file 2 [file Presentation_1.PDF]

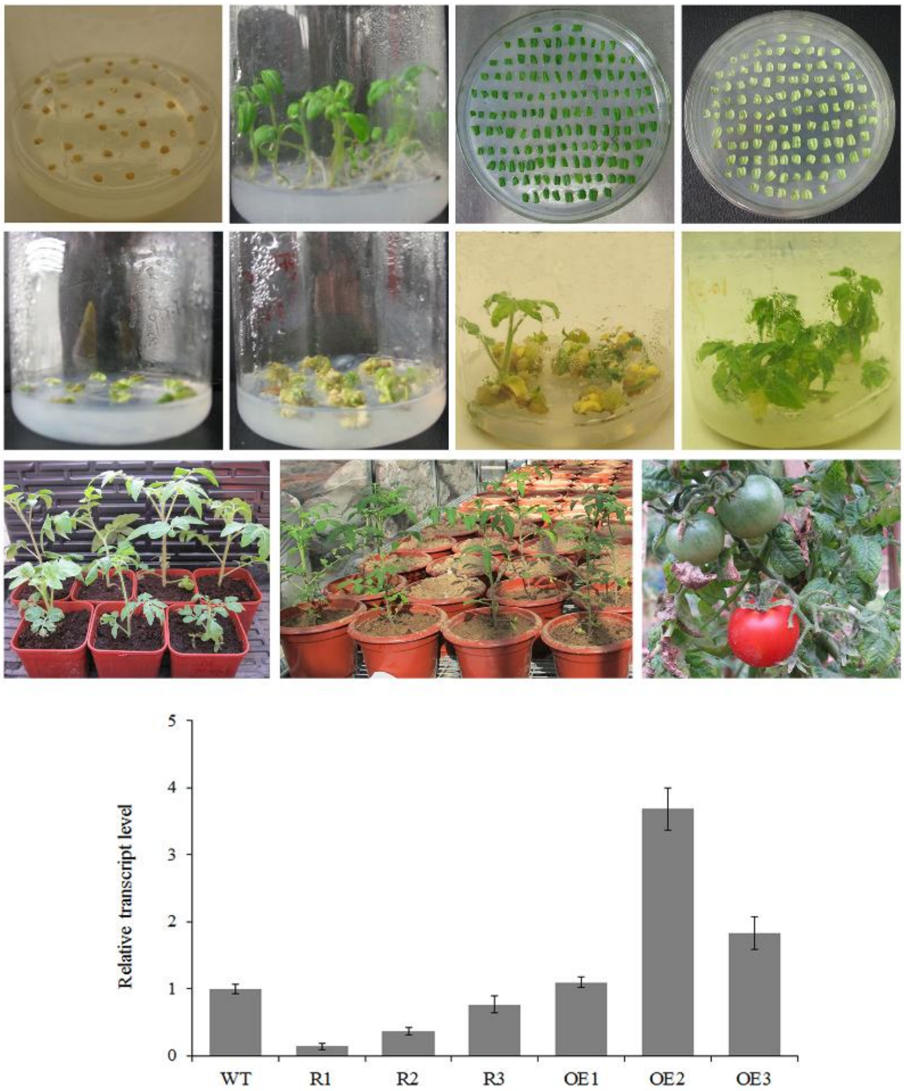

Supplementary Fig. S1 Workflow of tomato transformation and the expression level of *SlTDT* in WT, OE and RNAi transgenic lines. Tomato actin gene was used for normalization. Data represent the means ± SD of three replicates.

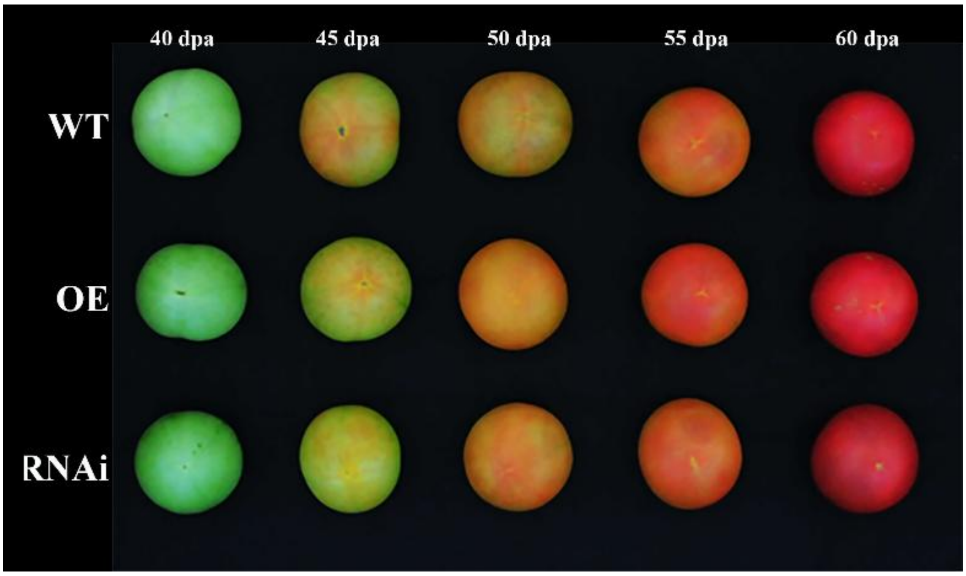

Supplementary Fig. S2 Tomato fruit at various ripening stages of WT, OE and RNAi transgenic plants. The ripening stages of tomato fruits were divided according to days after anthesis (dpa).

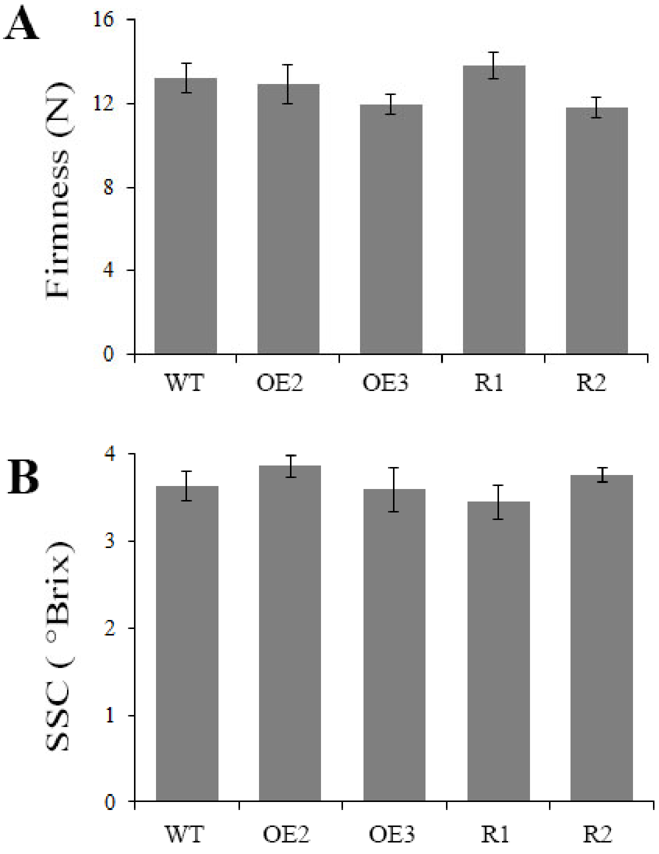

Supplementary Fig. S3 Quality attributes analysis of transgenic fruit. A, The total soluble solid contents (SSC) of tomato fruits from WT, OE and RNAi transgenic plants. B, Flesh firmness of tomato fruits from WT, OE and RNAi transgenic plants. Fruit firmness was determined by measuring compression using a penetrometer with a 8 mm diameter probe to a vertical depth of 1 mm on opposite sides of orange ripe fruits. Bars represent means ± SD of 9 ripen fruits.
